# Individual differences in the empathic experience of pain: An EEG and machine learning approach

**DOI:** 10.3758/s13415-025-01382-1

**Published:** 2026-01-08

**Authors:** Célia F. Camara, Sebastian Halder, Carina C. J. M. de Klerk, Alejandra Sel

**Affiliations:** 1 Centre for Brain Science, Department of Psychology, University of Essex, Colchester, UK; 2 School of Psychological Sciences, Birkbeck, University of London, London, UK; 3 School of Computer Science and Electronic Engineering, University of Essex, Colchester, UK

**Keywords:** EEG, Machine learning, Callous-unemotional traits, Vicarious pain, Empathy

## Abstract

Observing pain in others often elicits vicarious responses commonly considered as indices of empathy. However, the extent to which these responses reflect genuine empathic engagement remains subject of debate, with little research on how they may vary among individuals exhibiting low empathy traits like callousness or emotional detachment. To investigate this, we recorded EEG activity from 37 healthy participants to determine if neural responses to second-hand pain correlate with self-reported empathy and callousunemotional traits, while further testing the predictive utility of these signatures using single-trial machine learning classification. Although painful stimuli elicited distinct responses at the group level – specifically larger late positive potentials (LPP; 500–900 ms) and decreased theta and alpha power (650–1300 ms) over centroparietal brain regions –, machine learning classification of pain versus no-pain trials did not exceed chance accuracy, suggesting weak or heterogeneous neural differentiation at the single-trial level. Furthermore, pain-related EEG activity did not correlate with subjective pain ratings or empathy. Instead, the data revealed that callous and uncaring traits predicted attenuated LPP amplitudes, and unemotional traits were associated with stronger theta desynchronisation. Together, these findings suggest that neural markers of vicarious pain do not necessarily index empathic engagement but rather seem to reflect individual differences in emotional sensitivity.

**Supplementary Information:**

The online version contains supplementary material available at 10.3758/s13415-025-01382-1.

## Introduction

Empathy broadly refers to the ability to understand and share the emotions or feelings of others, which is often difficult to evoke in experimental settings (Zaki & Ochsner, 2012). One widely used approach has been through paradigms that elicit vicarious pain, in which images of nociceptive events directed at others – such as limbs in harmful situations or painful facial expressions – are presented (Coll, 2018; Jauniaux et al., 2019). These paradigms typically recruit perceptual and emotional processes essential for empathic responding (Bird & Viding, 2014a, b). In particular, they reveal that observing, imagining or merely anticipating pain in others engages emotional and somatosensory neural pathways overlapping with those involved in direct experiences of pain (Banissy & Ward, 2007; Decety & Meyer, 2008; Fallon et al., 2020; Lamm et al., 2011; Lockwood, 2016; Singer et al., 2004; Vachon-Presseau et al., 2012). These processes can be captured with high temporal resolution via electroencephalography (EEG).

EEG research shows that observing others in pain generally elicits positive event-related potentials (ERPs) amplified over centroparietal electrodes – including the P3 component (~300 ms) and the late positive potential (LPP, ~400–1,000 ms) – which are thought to index sustained attention and evaluative processing of socio-affective cues (de Vignemont & Singer, 2006; Fabi & Leuthold, 2017; Fan & Han, 2008). Beyond ERPs, second-hand pain observation has been associated with changes in oscillatory dynamics in alpha (8–12 Hz) and beta (13–30 Hz) power over centroparietal and sensorimotor regions (Chen et al., 2023; Cheng et al., 2008; Fabi & Leuthold, 2018; Lübke et al., 2020; Motoyama et al., 2017; Mu et al., 2008; Perry et al., 2010; Riečanský et al., 2015; Valentini et al., 2012). This phenomenon of event-related desynchronisation (ERD) in the alpha and beta band closely mirrors the neural dynamics observed during first-hand pain (Ploner et al., 2006; Riečanský et al., 2015; Whitmarsh et al., 2011). In particular, sensorimotor alpha desynchronisation – or *mu suppression* (Kuhlman, 1978) – has been associated with the perceived intensity of others’ pain (Babiloni et al., 2006; Hoenen et al., 2015), while beta suppression is thought to reflect motor resonance during pain observation (Harjunen et al., 2022; Riečanský et al., 2015). Furthermore, research has shown correlations between self-reported unpleasantness of observed painful stimuli and increases in theta power (3–5 Hz) over parietal regions (Mu et al., 2008) – paralleling responses to direct pain and tactile stimulation (Michail et al., 2016). Together, these EEG signatures offer insight into how individuals resonate with the affective states of others, and as such, they are often regarded as neurophysiological indices of empathic processing (Bird & Viding, 2014a, b).

Nevertheless, inconsistencies in the literature raise important questions about whether changes in EEG activity during pain observation can be attributed to empathy. While some studies have found significant correlations between pain-related changes in EEG activity and self-reported dispositional empathy (Cheng et al., 2008; Corbera et al., 2014; Fabi & Leuthold, 2017; Gonzalez-Liencres et al., 2016; Lübke et al., 2020; Vaes et al., 2016), others have failed to replicate these effects (Chen et al., 2023; Cogoni et al., 2023; Fabi & Leuthold, 2018; Perry et al., 2010; Van Dongen et al., 2018; Yang et al., 2009). For example, one study found that mu suppression did not correlate with empathy scores but was instead influenced by participants’ mood (Li et al., 2017). Similarly, a study comparing fibromyalgia patients with healthy controls found group differences in EEG responses during second-hand pain observation but no significant correlations with trait empathy in either group (de Tommaso et al., 2019). Moreover, while previous research has reported a significant correlation between trait empathy and pain-related ERPs (Fabi & Leuthold, 2017), a recent systematic review reports no significant associations between empathy scores and ERP components in empathy-related tasks (Almeida et al., 2024). These discrepancies underscore the need for further investigation into how electrophysiological responses to observed pain relate to an individual’s capacity for empathy.

One line of research that has gained increasing attention concerns the role of callous-unemotional traits in pain processing. Callous-unemotional traits comprise relatively stable socio-affective tendencies including emotional insensitivity, lack of guilt and remorse, and general disregard for others’ feelings and experiences (Frick & White, 2008). While predominantly observed in adults with psychopathy and at-risk youths, these traits also manifest subclinically in community samples (Byrd et al., 2013), providing valuable insights into how affective dispositions may vary across individuals. Theoretically, the expression of callous-unemotional traits signals deficits in affective and motivational processes that limit an individual’s capacity to resonate with others’ distress (Bird & Viding, 2014a, b). For instance, individuals exhibiting higher levels of callousness and shallow affect tend to underestimate both the intensity of distress and the severity of pain (Kaseweter et al., 2022). Additionally, individuals with high levels of these traits are typically less willing to engage in prosocial or helping behaviours when faced with another person in pain or need (Eisenberg & Miller, 1987; see, e.g., Sakai et al., 2016). Together, these findings suggest that callous-unemotional traits are closely tied to a reduced capacity for affective resonance, providing a more targeted lens for examining individual differences in vicarious pain processing than broader measures of dispositional empathy.

From a neurobiological standpoint, these behavioural responses are thought to arise from disruptions in neural mechanisms that enable vicarious pain (Bird & Viding, 2014a, b). Consistent with this view, evidence from EEG research shows that individuals higher in callous-unemotional traits typically show lower neural differentiation for pain versus no-pain stimuli. For instance, a study conducted by Decety et al. (2015) showed that, in a community sample of young adults, traits of callousness and shallow affect correlated with reduced differences in LPP amplitude to painful versus neutral stimuli – particularly when participants were instructed to focus on concern for others. More recent work similarly reports that traits of callousness, such as psychopathic meanness, predict lower LPP amplitudes in both pain perception tasks (Branchadell et al., 2024; Brislin et al., 2022) and in response to perceived harm to others (Van Dongen et al., 2018). Notably, participants with higher levels of these traits also reported lower perceived pain intensity under both self- and other-perspective conditions, suggesting that callous-unemotional traits may not only predict diminished affective responsiveness to others’ pain but also greater pain tolerance (Brislin et al., 2022).

Despite evidence for reduced neural differentiation, the relationship between callous-unemotional traits and electrophysiological responses to observed pain also show patterns that challenge the interpretation of EEG markers as direct indices of vicarious pain. Notably, studies in both criminal (Cheng et al., 2012) and community (Decety et al., 2015) samples exhibiting callous traits have reported increased mu suppression to second-hand pain – a neural signature typically interpreted as enhanced sensorimotor resonance and empathy (Mu et al., 2008). While these effects are not consistent across studies (Van Dongen et al., 2018), they nonetheless highlight the complexity of interpreting neural responses to others’ pain. Importantly, authors caution this enhanced mu suppression most likely reflects attentional engagement or cognitive monitoring of the painful stimulus or action rather than increased empathy or affective sharing (Cheng et al., 2012; Decety et al., 2015). This implies that EEG dynamics traditionally linked to vicarious pain also capture broader perceptual or attentional processes, which further complicates their interpretation as direct markers of vicarious pain and empathic processing.

Advances in machine learning provide new opportunities for addressing key methodological limitations inherent in conventional EEG analysis. EEG averaging methods, though effective at reducing noise, inevitably obscure trial-to-trial fluctuations that may capture meaningful variations in pain processing. By contrast, machine-learning methods can model EEG data at the single-trial level, capturing the full spatiotemporal and spectral complexity of the signal to detect more subtle and distributed patterns than mean amplitude or power difference metrics (Dinh et al., 2019; Mari et al., 2022). By enabling classification of pain versus no-pain conditions on a trial-by-trial basis, this approach provides a more fine-grained and rigorous assessment of the reliability and specificity of pain-related neural responses within and across individuals. Consequently, single-trial classification offers a promising avenue for characterising the neural dynamics underpinning pain processing and advancing the field beyond traditional averaging.

Empirical evidence supports the effectiveness of machine-learning techniques in decoding neural responses to pain. For instance, recent studies by Mari et al. (2023a, b) demonstrated that machine learning models can reliably distinguish time-frequency features associated with direct pain experiences, achieving above-chance classification accuracies ranging from 58% to 73%. Building on this work, Wang et al. (2025a, b) applied similar approaches to EEG data collected during the observation of others’ pain. Their findings revealed that machine learning could also differentiate ERP signals corresponding to pain versus no-pain trials, with classification accuracy notably improving when participants underwent a guilt induction manipulation. This suggests that feelings of guilt can increase the neural distinctiveness of second-hand pain processing, thereby improving the model’s ability to detect relevant EEG features. As such, it is possible that individuals with reduced sensitivity to such emotions – like those high in callous-unemotional traits – display weaker neural differentiation between painful and neutral stimuli, which further highlights the relevance of these traits for examining individual variability in pain processing.

A critical limitation in Wang et al.’s study, however, is that classification performance was not formally tested against chance, raising concerns about whether the observed improvements truly reflect enhanced neural specificity or are simply due to random fluctuations. Supporting this concern, studies using both passive pain observation (Mari et al., 2023c) and active paradigms (Mari et al., 2025) found that classifiers trained on EEG data fail to exceed chance-level accuracy, indicating a lack of reliability and consistency in EEG responses to second-hand pain. In fact, classifiers were consistently more sensitive to visual features of the stimuli (e.g., distinguishing scenes from faces) than to neural activity specifically associated with the perception of others’ pain. These findings contribute to the ongoing uncertainty about whether EEG responses to observed pain genuinely reflect socio-affective processing or are primarily driven by perceptual and attentional factors. They highlight the need for systematic replication using chance-level testing to determine whether machine learning can reliably classify EEG signals associated with second-hand pain.

In the current study, we examine how neural responses to second-hand pain might vary as a function of individual differences in socio-affective traits, including empathy and callous-unemotional traits. We recorded EEG activity during a passive viewing task in which participants watched painful and neutral scenarios involving human targets. To capture trait-level variability, we assessed participants’ subjective perceptions of the pain experienced by the targets, whereas empathy and callous-unemotional traits were assessed via self-report questionnaires. Because callous-unemotional traits index a more direct disruption of socio-affective processing than general empathy measures, we hypothesised that higher callous-unemotional scores would correlate with reduced differentiation of the P3 and LPP during pain versus no-pain conditions (Brislin et al., 2022; Decety et al., 2015). We did not make directional predictions regarding oscillatory dynamics given the mixed evidence in prior work (Decety et al., 2015; Van Dongen et al., 2018).

Furthermore, we employed machine-learning techniques to classify EEG data at the single-trial level. Building on previous research that has primarily examined ERP features for classification (Mari et al., 2023c, 2025), we extended this work by also including time-frequency classifiers. Our goal was twofold:

1. To evaluate whether the robust classification accuracy reported in direct pain studies (Dinh et al., 2019; Mari et al., 2022, 2023a, b, c) generalises to second-hand pain, and
2. To test whether classifier performance might be influenced by individual differences in empathy and callous-unemotional traits.

Importantly, correlations with trait measures were contingent on classifiers performing above chance levels, ensuring that any observed trait-related associations reflected meaningful neural discriminability rather than random fluctuation.

## Method

This study was conducted in accordance with the ethical standards of the Declaration of Helsinki and was granted approval by the University of Essex Science and Health Ethics Sub-committee (Ethics approval number: ETH2122-0281). All scripts used for the EEG and machine learning analyses are available at the Open Science Framework (OSF) repository: https://osf.io/f2p9b/?view_only=076befab2730484ca1257d2eb7939d29.

### Participants

Participants were recruited from both university and community samples through word of mouth, email invitations, and the University of Essex’s participant recruitment platform. Sample selection was limited to right-handed young adults, aged 18–45 years, with normal or corrected-to-normal vision and no history of major neurological conditions. Initially, 50 respondents volunteered to take part. However, four withdrew before completing the experiment, eight were excluded due to significant data contamination (e.g., excessive ocular or motion artefacts in the EEG signal), and one participant who was primarily left-handed was also excluded. This resulted in a final core sample of 37 participants for the primary analyses, including 20 men and 17 women aged 20–35 years (*M* = 24.49, *SD* = 3.15 years).

After completing a pre-screening survey online, eligible participants completed a second online survey (~15 min), followed by a lab session (~2–3 h). However, survey data were missing for one participant, leaving a total of 36 participants (19 men) for trait-level assessments. All participants provided their written informed consent prior to participation and were reimbursed with a £10 voucher upon completion of the experiment.

### Study design and measures

#### Pre-screening

We first assessed participants’ handedness via the Edinburgh Handedness Inventory questionnaire (Oldfield, 1971). Only participants with a positive laterality quotient (LQ) – classified as right-handers (McMeekan & Lishman, 1975) – were included in the study to maintain consistency within our sample and align with prior research indicating lateralisation in pain perception (e.g., Hofman & Schutter, 2012; Timmers et al., 2018). An additional pre-screening step included checking for participants’ age before proceeding to the main survey.

#### Online survey

In a second online survey, we assessed participants’ baseline levels of empathy and callous-unemotional traits via self-report questionnaires. Trait empathy was evaluated using the *Affective and Cognitive Measure of Empathy* (ACME; Vachon & Lynam, 2016). This scale includes items to measure cognitive empathy (e.g., “I can usually tell how people are feeling”), affective resonance (e.g., “I feel awful when I hurt someone’s feelings”), and affective dissonance (e.g., “It’s funny to see people get humiliated”). Items in the ‘affective dissonance’ subscale were reverse-coded, such that higher scores represent higher levels of empathy (Vachon & Lynam, 2016). For callous-unemotional traits, we used the *Inventory of Callous Unemotional Traits* (ICU; Frick et al., 2003), which includes items examining traits of callousness (e.g., “The feelings of others are unimportant to me”), uncaring behaviours (e.g., “I try not to hurt others’ feelings”, reverse coded), and unemotional traits (e.g., “I express my feelings openly”) (Decety et al., 2012; Goubert et al., 2005).

#### Lab experiment

**Fig. 1:**
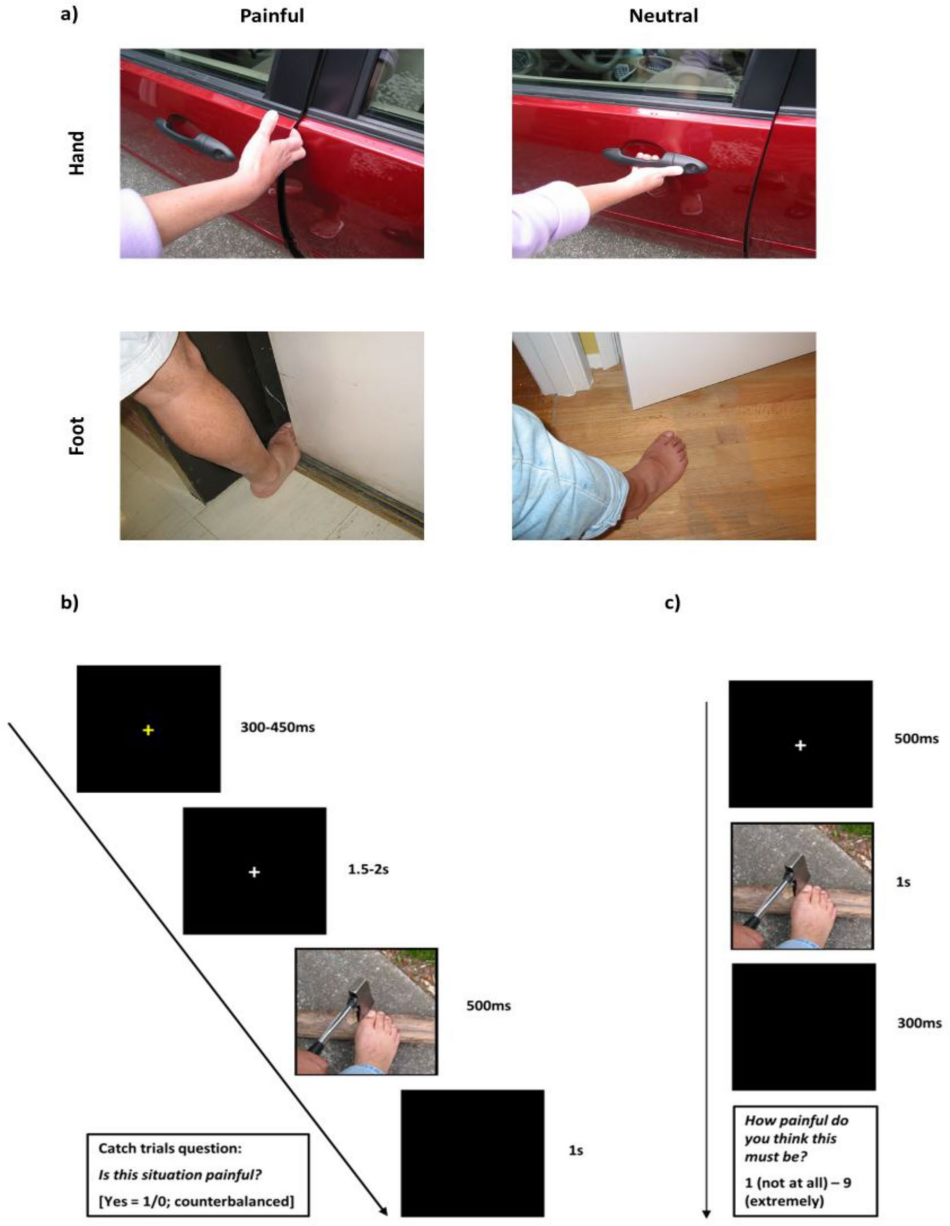
Visual example of (**a**) images per limb and pain condition; (**b**) the experimental design for the passive viewing task, including the catch trials question; and (**c**) the experimental design for the rating task, including the pain intensity question and rating scale

Within 1 week of completing the online survey, participants attended a lab session at the University of Essex’s Centre for Brain Science. Upon arrival, they completed a brief safety screening to confirm eligibility for EEG recording and task participation. The experiment consisted of two computer-based tasks programmed in *E-Prime* (version 2, Psychology Software Tools, Inc.) and followed a 2 × 2 factorial design. Stimuli comprised first-person perspective images depicting identical actions that implied either physical pain or no pain to human hands or feet, adapted from Jackson et al. (2006; see Fig. 1a). The experiment was conducted in a dimply lit, sound-attenuated room, with participants seated approximately 50 cm from the display monitor. EEG electrodes were applied and impedances kept below 50 Ω prior to task onset.

The first task consisted of a *Passive Viewing* paradigm (Fig. 1b), which required participants to observe the images while imagining the level of pain experienced by the depicted individual. This task aimed to measure neural responses to others’ pain under conditions of minimal cognitive interference. A total of 200 trials were presented in four blocks of 50, with each trial beginning with a yellow fixation cross (300–450 ms) – prompting participants to blink in order to minimise ocular artefacts – followed by a white fixation cross (1.5–2 s) signalling the start of a new trial. Images were presented for 500 ms in a random order. On 20% of trials (catch trials), participants indicated whether the scenario was painful by pressing ‘1’ or ‘0’ on a keyboard (response keys counterbalanced), though EEG data from these trials were excluded to avoid decision- or motor-related confounds. Participants then completed a *Pain Rating* task (Fig. 1c), in which they provided their subjective evaluations of pain intensity for each image on a 9-point Likert scale. This task included a total of 64 trials (32 per condition), equally distributed across four blocks. Each trial began with a 500-ms fixation cross, followed by a 1-s image presentation and a rating period 300 ms later. EEG was not recorded during this task, though the cap was only removed when the session was completed.

### EEG procedure

#### Data acquisition

EEG activity was recorded using an elastic EEG cap equipped with 60 active scalp electrodes. Electrode placement followed the 10 M equidistant layout (EasyCap GmbH, 2018). To ensure precise positioning, anatomical landmarks (pre-auricular points, nasion, and inion) were used to locate the head centre, referenced to the Cz electrode. The ground electrode was placed at AFz, and electro-oculogram (EOG) electrodes were positioned above and below the left eye to monitor ocular artifacts. All electrodes were online-referenced to the left mastoid. EEG data were recorded using BrainAmp amplifiers (BrainProducts, Munich, Germany) with a 0.1 μV analogue-to-digital conversion and a sampling rate of 1,000 Hz.

### Data analysis

Data analysis was conducted using custom MATLAB scripts with functions from the Fieldtrip toolbox (Oostenveld et al., 2011).

#### Preprocessing

The raw EEG signal was band-pass filtered between 0.1 and 30 Hz, and downsampled to 500 Hz. The signal was subsequently segmented according to pain (painful vs. neutral) and limb (hand vs. foot) conditions, with epochs spanning from −1.5 s before to 2 s after stimulus onset. Although the experiment was originally designed to include 200 trials (40 main trials and ten catch trials per condition), the final dataset comprised an average of 159.54 trials (*SD* = 4.13). Post hoc analysis indicated that this reduction was non-significant (*p* =.290), with most participants retaining ≥50 trials per condition (see Electronic Supplementary Material (ESM) Fig. 1). After segmentation, epochs exceeding ± 100 µV in any channel were removed by automated methods, supplemented by visual inspection. Independent Component Analysis (ICA) further filtered out physiological artefacts (e.g., eye blinks, saccades, heartbeat, or muscle activity), with an average exclusion of three components removed per participant (*SD* =.77; ESM Table 1). Noisy data in specific trials were addressed by interpolating from neighbouring electrodes, though trials were manually excluded if more than five channels required interpolation. Data were then re-referenced to the average of all scalp electrodes, excluding non-scalp sites.

#### Feature extraction

To characterise neural responses in both the time and time-frequency domains, we conducted ERP and spectral power analyses on the pre-processed EEG data. For ERP analyses, signal was baseline-corrected using a ˗500- to 100-ms pre-stimulus interval, and individual ERPs were averaged per condition across participants. ERP topographies were examined by averaging voltage distributions across electrode sites time-locked to stimulus onset (500–1,000 ms post-stimulus). For spectral power analyses, we used a multitaper transformation to individual epochs across 4–30 Hz (in 1-Hz steps), converting time-domain EEG signals into the frequency domain using a fast Fourier transform (FFT) with a Hanning taper (three cycles) and zero-padding to enhance spectral resolution and computational efficiency (Goldfine et al., 2011). Relative baseline correction was applied within −1.1–0 s before stimulus onset. In both analyses, statistical significance was evaluated using a nonparametric randomisation approach with 5,000 iterations (Maris & Oostenveld, 2007) to identify clusters of significant activity across time, space, and frequency relative to baseline. ERP results revealed distributed activity across frontal, central, centroparietal, and parieto-occipital regions, while spectral analyses showed similar patterns of activation in frontal, centroparietal, and parieto-occipital regions. Electrodes within these regions were subsequently grouped into regions of interest (ROIs) for further statistical analysis.

#### Cluster-based permutation

To identify significant effects in both time and time-frequency domains, we employed a nonparametric, within-subject permutation-based approach. This involved computing repeated-measures *t* tests with 5,000 random permutations for each comparison between experimental conditions at every time point (Maris & Oostenveld, 2007). Contiguous data points exceeding the significance threshold (*p* <.05) were grouped into clusters. The sum of *t* values within each cluster was used as the cluster-level statistic, which was then compared against the permutation distribution to determine significance. To control for multiple comparisons, we applied stepwise Bonferroni-Holm correction.

#### Machine learning classification

We performed binary classification analyses to evaluate the signal-trial discriminability between painful and neutral scenarios, comparing ERP versus time-frequency features. Analyses were implemented in Python (version 3.13.2) using MNE-Python (version 1.9.0) and scikit-learn, running on a MacBook Pro (M2 Max, 96 GB RAM, Sequoia 15.5).

For ERP classification, epochs were downsampled to 20 Hz, cropped to 0–1.2 s post-stimulus, vectorised and normalised using Min-Max scaling prior to model training. For time-frequency classification, we applied a bandpass filter across six frequency bands – theta (4–8 Hz), alpha (8–12 Hz), low beta (12–16 Hz), mid beta (16–20 Hz), high beta (20–24 Hz), and low gamma (24–30 Hz) – followed by a Principal Component Analysis (PCA) dimensionality reduction to 60 components, and a Common Spatial Pattern (CSP) analysis, retaining four components per band. The features used for classification were the log-variance of the resulting CSP components. To prevent data leakage, CSP fitting was performed exclusively on training data within each cross-validation fold.

Classifier performance was estimated at the individual-participant level using fivefold stratified cross-validation across five algorithms:

- Linear Discriminant Analysis (LDA) with automatic shrinkage
- Support Vector Machine (SVM) with an RBF kernel,
- Random Forest (RF) (100 estimators),
- XGBoost, and
- Gaussian Naive Bayes (GNB).

Each model was tested under three feature selection schemes:

1. No selection,
2. SelectKBest retaining the top 100 features, and
3. L1-embedded selection (using L1-penalised logistic regression with liblinear solver and 1,000 maximum iterations).

Classification accuracy was evaluated against chance using the binomial cumulative distribution thresholding method described by Combrisson and Jerbi (2015), with the following function:

$$Statistical Threshold=binoinv\left(1-a,n,\frac{1}{c}\right)\times \frac{100}{n}$$

Using this function in MATLAB, we determined that for a binary classification (*c* = 2) with ~160 trials (*n*) per participant and a significance level of *α* =.05, a classifier had to achieve an accuracy of ≥ 56.25% to be considered reliably above chance.

## Results

### Sample characteristics

Survey scores were analysed on SPSS (IBM SPSS Statistics for Windows, Version 29.0). Overall, participants’ average scores in empathy (ACME) and callous-unemotional traits (ICU) scales fell within the estimated normative range established in previous research (Byrd et al., 2013; Dryburgh & Vachon, 2019). Descriptive statistics for total and subscale scores are presented in Table 1.

**Table 1:** Summary of sample characteristics

	All			Men			Women		
*Variables*	*n*	*M*	*SD*	*n*	*M*	*SD*	*n*	*M*	*SD*
Age, y	37	24.49	3.11	20	25.25	2.34	17	2.59	3.71
Ratings	37	4.59	.94	20	4.2	1.29	17	5.07	.28
RTs, ms	37	585.41	663.22	20	404.09	532.15	17	776.95	757.33
Understanding	36	39.94	5.01	19	39.37	5.29	17	40.59	4.74
Resonance	36	51.94	5.13	19	50.53	5.18	17	53.53	4.72
Dissonance	36	44.86	3.23	19	44.53	3.19	17	45.24	3.35
ACME total	36	136.75	9.29	19	134.42	9.24	17	139.35	8.90
Callousness	36	6.06	2.99	19	7.16	2.67	17	4.82	2.92
Uncaring	36	5.50	2.50	19	6.26	2.18	17	4.65	2.62
Unemotional	36	7.50	2.95	19	7.95	2.55	17	7.00	3.35
ICU total	36	19.06	6.39	19	21.37	4.75	17	16.47	7.11

*n* = number of participants, *M* = mean, *SD* = standard deviation, RTs = rating times

Ratings and RTs indicate the difference in participants’ perceived pain intensity and rating times in pain vs. no-pain conditions. Scores are presented for the ACME (Affective and Cognitive Measure of Empathy) and the ICU (Inventory of Callous-Unemotional Traits) scales. Additional data separating men and women are also provided for gender comparisons

### Subjective pain perception

**Fig. 2:**
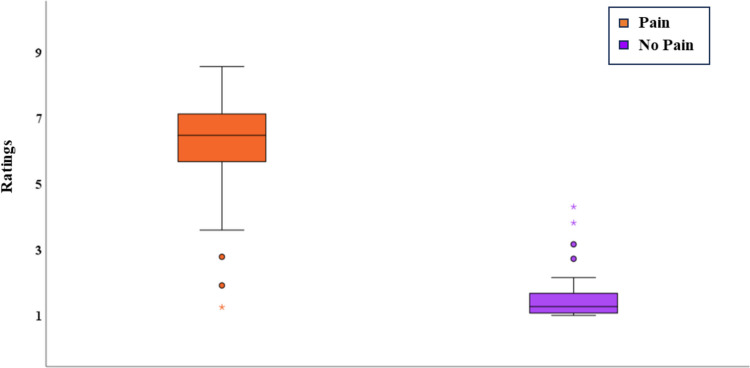
Picture ratings in painful and neutral conditions per participant. Pain intensity was rated from 1 (no pain) to 9 (extreme pain). As illustrated, on average, each participant assigned moderate to high pain ratings to images depicting painful scenarios (**orange box**), whereas non-painful stimuli (**purple box**) were mostly rated as not painful

Participants accurately distinguished painful from non-painful scenarios in 79.3% of catch trials (*SD* = 11.6%), confirming the reliability of our experimental design. On average, painful images were rated as moderately painful (*M* = 6.15, *SD* = 1.73), whereas neutral images were predominantly rated as not painful (*M* = 1.70, *SD* =.99). Follow-up *t* tests confirmed that this difference was significant, *t*(36) = 16.16, *p* <.001, Cohen’s *d* = 2.66. Participants also took longer to evaluate painful images than non-painful ones, *t*(36) = 5.28, *p* <.001, Cohen’s *d* =.87 (average reaction times are given in Table 1). However, as illustrated in Fig. 2, there was substantial variability in pain ratings, with scores ranging from 1.25 to 8.56 on a 9-point scale.

### EEG correlates of perceived pain

**Fig. 3:**
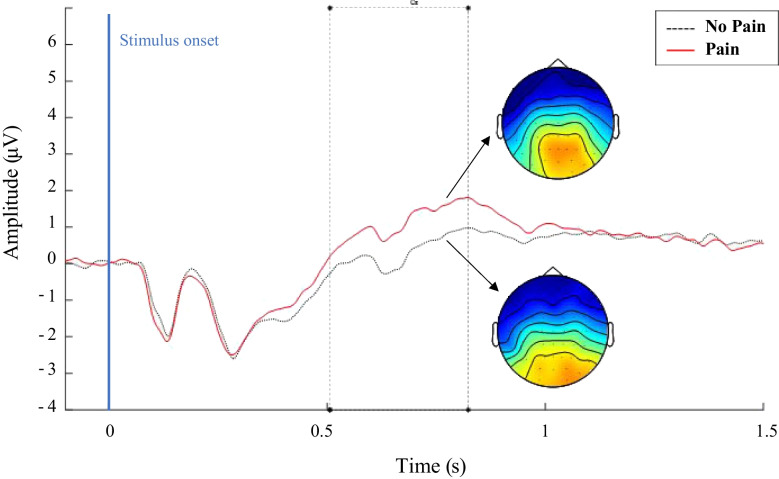
Grand average event-related potential (ERP) at Cz for painful vs. neutral images, showing a late positive potential with larger amplitude during painful as compared with the neutral condition. Significant time range (0.508–0.918 s) is highlighted in the black dotted rectangle

Consistent with our hypotheses, painful images elicited larger LPP amplitude compared to non-painful stimuli, with maximal distribution over central, *t*(36) = 1.15, *p* <.001, parietal, *t*(36) = 2.82, *p* =.004, and centroparietal, *t*(36) = 3.10, *p* =.002, electrode clusters (C1, C2, C4, Cz, CP1, CP2, CP3, CP4, CPz, P1, P2, P4, Pz, POz) between 508 and 918 ms after stimulus onset (see Fig. 3 for an example). To examine whether this neural response reflected perceived pain intensity, we correlated mean LPP amplitudes with participants’ subjective pain ratings. This analysis revealed no significant association, as indicated by large 95% confidence intervals (CIs) including zero (Amrhein et al., 2019; see data in ESM Table 2). This suggests that the observed LPP enhancement was not modulated by participants’ subjective evaluations of pain severity.

**Fig. 4:**
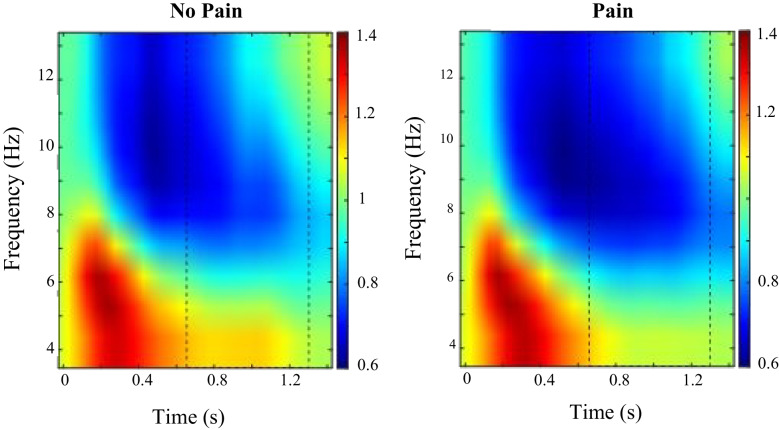
Grand-averaged time-frequency across centroparietal region per pain condition. Electrode sites include: C5, C3, C1, Cz, C2, C4, C6, TP7, CP5, CP3, CP1, CPz, CP2, CP4, CP6, TP9 and T7. The black rectangle represents the time frequency window (650–1,300 ms) in which changes in spectral power in the alpha and theta frequencies are observed

In the time-frequency domain, pain observation was associated with significant decreases in theta, *t*(36) = −3.06, *p* =.004, and alpha power, *t*(36) = −2.68, *p* =.011, showing maximal distribution over centroparietal electrodes (C5, C3, C1, Cz, C2, C4, C6, TP7, CP5, CP3, CP1, CPz, CP2, CP4, CP6, TP9, T7) between 650 and 1,300 ms after stimulus onset (Fig. 4). However, consistent with the LPP findings, these changes in oscillatory power did not correlate with participants’ subjective pain ratings (ESM Table 2), indicating that oscillatory responses were likewise not strongly related to individual variability in perceived pain intensity. No significant effects were observed in the beta band frequency range.

### Correlations with empathy and callous-unemotional traits

Correlations with trait measures used the ACME and ICU subscale scores. A negative correlation was observed between callousness scores and pain-related ERP amplitude over centroparietal electrodes, r = -.34, 95% CI [-.60, -.02]. Similarly, uncaring behaviour showed negative correlations with ERP amplitude changes over both centroparietal, r = -.36, 95% CI [-.61, -.03], and central electrode sites, r = -.34, 95% CI [-.60, -.01]. These findings indicate that diminished socio-affective engagement, as reflected in higher callousness and uncaring traits, corresponds to attenuated ERP responses typically associated with the cognitive and affective evaluation of observed pain. In contrast, unemotional trait scores positively correlated with theta power modulation over centroparietal electrodes, r =.39, 95% CI [.07,.64], indicating increased theta desynchronisation during pain versus no-pain conditions among participants reporting lower emotional expressivity. None of these traits, however, correlated with participants’ subjective ratings of pain intensity during the task (see data in ESM Table 2).

### Classification performance

The machine-learning classification analysis provided no evidence for reliable single-trial discrimination between painful and neutral scenarios. Across all tested algorithms and feature selection strategies, classification accuracy consistently remained below the predefined chance-level threshold of 56.25%. A detailed examination of modelling parameters showed that classifier choice (LDA, SVM-RBF, Random Forest, XGBoost, Naive Bayes) and feature selection methods (no selection, SelectKBest, and L1-embedded) had minimal impact on performance, achieving comparable outcomes (ESM Fig. 2). This lack of model sensitivity was particularly evident in the time-frequency domain, where no specific frequency band (4–30 Hz) provided superior discriminative information – with all spectral ranges clustering around chance level. Nevertheless, a modest overall advantage was observed for classifiers trained on ERP features compared with those using spectro-temporal features. In particular, the ERP LDA model without feature selection achieved the highest median accuracy (56.1%, σ^2^ = 0.16%), falling only marginally below the threshold of statistical significance.

A follow-up inspection of individual-level results revealed considerable variability in ERP classification performance, with accuracies in the best-performing model ranging from 40% to 70% across participants. Comparable patterns were observed for time-frequency classification, with accuracies ranging from 42% to 61%. Across participants, deviations from the group mean were roughly balanced, with about half performing above and half below the average accuracy, while performance consistency varied notably, with standard deviations ranging from.02 to.07. The results of this analysis are illustrated in ESM Fig. 3.

Overall, these findings suggest that neural signals differentiating pain from no-pain conditions were either too weak or too heterogeneous to be reliably detected using the present group-level classification approach. Consequently, the planned analyses examining associations between single-trial neural discriminability and participants’ empathy and callous-unemotional traits were not conducted.

## Discussion

The present study examined how individual differences in empathy and callous-unemotional traits shape the neural processing of vicarious pain, combining EEG with machine-learning classification to assess the consistency and predictive reliability of pain-evoked neural responses. Replicating findings from previous studies (Fabi & Leuthold, 2017; Fan & Han, 2008; Mu et al., 2008), we found that pain observation was associated with increased LPP amplitudes and a delayed reduction in theta and alpha power over centroparietal regions – reflecting emotional evaluation and salience processing of pain-related cues (de Vignemont & Singer, 2006; Fabi & Leuthold, 2017; Fan & Han, 2008). Nevertheless, neither LPP amplitude nor oscillatory activity correlated with participants’ subjective pain ratings or self-reported empathy. These results align with growing evidence suggesting that EEG responses specific to second-hand pain observation do not necessarily reflect trait-level empathy (e.g., Chen et al., 2023; Fabi & Leuthold, 2018; Perry et al., 2010; Yang et al., 2009). In fact, measures of trait empathy typically assess stable, self-reflective dispositions, whereas EEG captures more rapid and transient neural dynamics. As such, pain-related EEG activity in these paradigms may be more related to perceptual and attentional processes triggered by salient social cues than with the higher-order cognitive and motivational components captured by broader empathy measures (Almeida et al., 2024; Coll et al., 2017).

In contrast, correlations with callous-unemotional traits revealed distinct and dissociable patterns in pain-related EEG activity. As hypothesised, higher levels of callousness and uncaring behaviour were associated with reduced LPP differentiation between painful and neutral stimuli, consistent with prior evidence linking psychopathic and callous traits to diminished LPP amplitudes during pain observation (Branchadell et al., 2024; Brislin et al., 2022; Decety et al., 2015). In our study, unemotional traits additionally correlated with pain-related theta desynchronisation, a response previously linked to cognitive appraisal of affective stimuli (Mu et al., 2008). These findings highlight the specificity of different callous-unemotional subcomponents in shaping the neural processing of others’ pain. By examining these subcomponents separately, the study suggests that traits reflecting emotional detachment and reduced concern for others (i.e., callous and uncaring traits) may predict diminished affective engagement with others’ pain, whereas high unemotional traits potentially involve compensatory recruitment of top-down control processes during pain processing. This pattern aligns with insights from psychopathy research proposing a dissociation between cognitive and affective empathy, where the ability to understand others’ mental states is preserved even when affective resonance is reduced (Seara-Cardoso et al., 2015). Although our sample was relatively small and comprised non-clinical participants, these findings suggest that subclinical variation in callous-unemotional traits can reveal subtle but theoretically meaningful differences in affective responsivity and motivational salience to second-hand pain stimuli.

From a broader perspective, this variability provides an interpretive context for our machine-learning findings, highlighting the complex and heterogeneous nature of neural responses to others’ pain. Notably, classification across ERP and time-frequency feature sets consistently revealed failure to exceed the predefined chance-level threshold. This outcome is consistent with recent studies equally failing to classify ERP signals to second-hand pain above chance levels (Mari et al., 2023c, 2025), indicating that the neural signals to second-hand pain are not reliably detected at the single-trial level. Extending this literature, our data demonstrate that these null findings are consistent across multiple algorithms and feature domains. Such convergence indicates that the observed effects are not algorithm-specific artefacts but instead reflect genuine variability in pain-related neural signatures. Indeed, classification accuracies varied considerably across participants, with some showing above-chance decoding and others performing near or below chance. In this sense, the failure to decode pain versus no-pain trials above chance does not necessarily imply the absence of discriminative neural information but rather its inconsistency across individuals and trials.

Such inconsistencies indicate that neural signatures of second-hand pain are likely subtle, temporally variable, and context dependent, which poses inherent challenges for single-trial decoding in conventional experimental paradigms (Mari et al., 2025). In particular, the use of static, decontextualised images – though advantageous for experimental control and temporal precision – may lack the emotional salience and ecological validity required to elicit strong, temporally coherent vicarious pain responses. In fact, while static depictions of body parts in painful scenarios tend to activate perceptual and attentional systems associated with visual processing, they may fail to elicit the dynamic affective resonance and social meaning intrinsic to real-life pain experiences (Wang et al., 2025a, b). As a result, the evoked neural responses were likely too weak and heterogeneous to support consistent single-trial decoding. Moreover, given that classifier performance improves with data volume (Gómez-Tapia et al., 2022; Rommel et al., 2022), the relatively small sample size in this study – combined with trial loss during artefact rejection – further reduced the effective number of training examples per participant, constraining model sensitivity.

To address these challenges, future studies should explore paradigms that more closely approximate naturalistic social perception. Incorporating dynamic stimuli (e.g., videos depicting facial and bodily expressions of pain) or immersive virtual reality environments could evoke more sustained and emotionally engaging responses, thereby enhancing both the amplitude and temporal stability of relevant neural signals. Building on recent findings by Wang et al. (2025a, b), integrating these paradigms with experimental manipulations that modulate socio-affective engagement (for instance, through guilt induction, perspective-taking prompts, or shared-goal contexts) could further increase the neural specificity and discriminability of second-hand pain representations. These manipulations might help disentangle the cognitive and affective components of empathic processing and clarify how motivational or moral contexts shape neural signatures of others’ suffering. Importantly, replicating and validating such findings with rigorous tests of classification performance and cross-validation procedures are essential for establishing their robustness and generalisability. Together, these methodological advancements could substantially refine our understanding of the neural mechanisms underlying second-hand pain processing, offering a more reliable and context-sensitive framework for interpreting electrophysiological indices of pain empathy.

## Conclusion

In summary, the current study demonstrates that while observing others’ pain elicits consistent neural responses, these responses do not straightforwardly reflect individual differences in trait empathy. Instead, callous-unemotional traits provide a more sensitive lens for examining variability in affective responsiveness and motivational salience during vicarious pain processing. Machine-learning analyses further highlighted substantial inter-individual and trial-level variability, underscoring the challenges of reliably decoding pain-related neural activity at the single-trial level. Future research should aim to replicate these effects with larger, more diverse samples and more ecologically valid paradigms to establish the reliability and specificity of EEG correlates of pain empathy.

## Supplementary Information

Below is the link to the electronic supplementary material.

Supplementary file1 (DOCX 331 KB)

## Data Availability

Available via the Open Science Framework (OSF) (https://osf.io/f2p9b/?view_only=076befab2730484ca1257d2eb7939d29)

## Code availability

Available via the OSF (https://osf.io/f2p9b/?view_only=076befab2730484ca1257d2eb7939d29)
