# Supplementary material for: Individual differences in the empathic experience of pain: An EEG and machine learning approach
Source: Cogn Affect Behav Neurosci. 2026 Jan 8;26(3):1237–50. doi: 10.3758/s13415-025-01382-1 (PMC13260166; doi:10.3758/s13415-025-01382-1)
Supplement: Supplementary file 1 — Supplementary file1 (DOCX 331 KB) [file 13415_2025_1382_MOESM1_ESM.docx]

**Supplementary Material**

Individual differences in the empathic experience of pain: An EEG and machine learning approach

Célia F Camara*^1^, Sebastian Halder^2^, Carina CJM de Klerk^1^, Alejandra Sel^1^

^1^*Centre for Brain Science*, *Department of Psychology, University of Essex*

^2^*School of Computer Science and Electronic Engineering, University of Essex*

**^*^Corresponding author:**

Célia F Camara

camaracelia@outlook.com


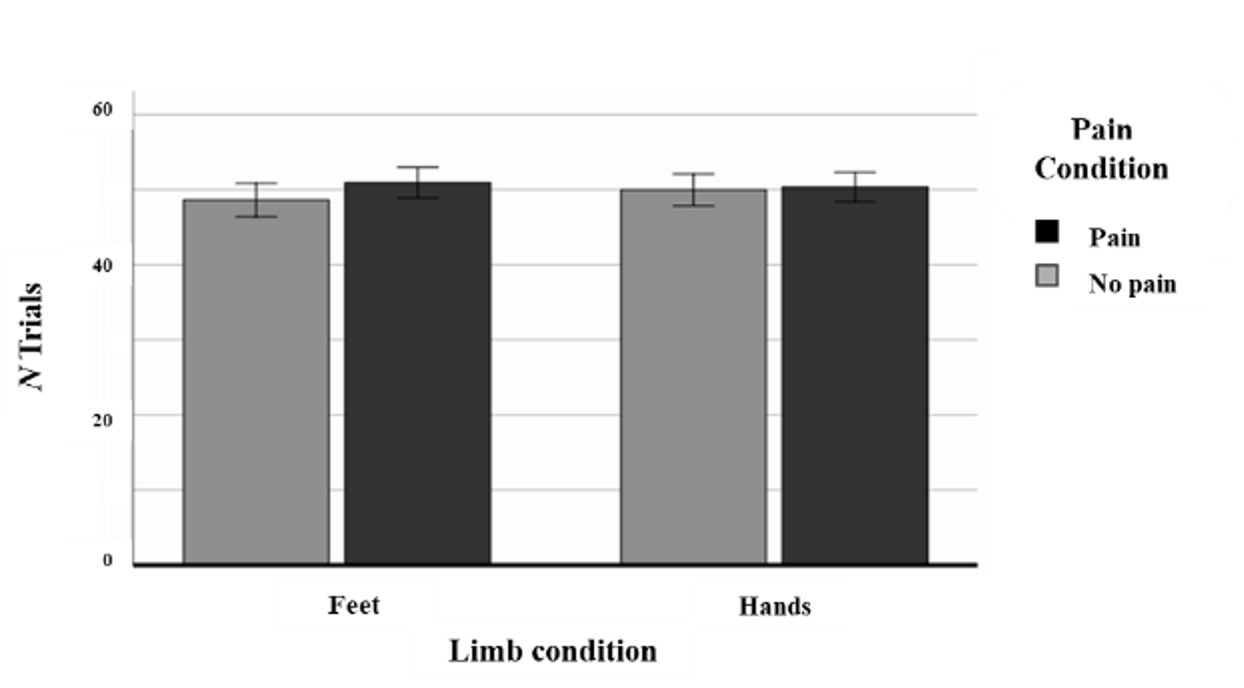
***Supplementary Figure 1*.** Averaged number of trials in painful (black) and neutral (grey) conditions during the passive viewing task. On average, participants completed approximately 50 trials per condition overall (including catch trials). Analysis of within-subject contrasts showed no significant difference in the number of trials between the different limb and pain conditions (F(1,39) = 1.15, p = .290).

Error bars represent ±2.5 standard error.

| **Participant** | **Initial trials** | **Components** | **Channels** | **Final trials** |
| --- | --- | --- | --- | --- |
| 1 | 160 | 2 | 'Cz', 'TP9' | 125 |
| 2 | 161 | 2 | 'Cz', 'T7', 'TP7' | 109 |
| 3 | 153 | 2 | 'TP9' | 109 |
| 4 | 156 | 4 | *None* | 124 |
| 5 | 162 | 3 | 'TP9', 'Cz' | 108 |
| 6 | 156 | 3 | 'T7', 'T8', 'O2', 'Cz' | 107 |
| 7 | 161 | 3 | 'O2', 'TP9', 'AF8', 'Cz' | 125 |
| 8 | 154 | 3 | *None* | 123 |
| 9 | 165 | 2 | 'CP2' | 136 |
| 10 | 158 | 4 | 'CP2', 'TP9', 'O2', 'P6' | 124 |
| 11 | 156 | 3 | 'TP8', 'FT8', 'TP10', 'AF8' | 132 |
| 12 | 163 | 3 | 'TP9' | 133 |
| 13 | 159 | 3 | 'TP9' | 137 |
| 14 | 161 | 4 | 'TP9', 'FP1' | 114 |
| 15 | 160 | 2 | 'Cz' | 137 |
| 16 | 169 | 3 | 'CP2', 'TP10', 'FT8', 'T7', 'FP1', 'AF7' | 125 |
| 17 | 156 | 4 | 'Cz', 'TP9' | 102 |
| 18 | 162 | 4 | 'T8', 'TP9' | 123 |
| 19 | 161 | 3 | 'Cz' | 109 |
| 20 | 157 | 2 | 'Cz' | 123 |
| 21 | 162 | 3 | 'CP2','TP9' | 127 |
| 22 | 156 | 3 | 'TP9' | 128 |
| 23 | 154 | 3 | 'TP9', 'T8' | 137 |
| 24 | 163 | 5 | 'FT7', 'AF8', 'AF7', 'F8' | 118 |
| 25 | 162 | 4 | 'TP9' | 140 |
| 26 | 160 | 3 | 'TP9' | 112 |
| 27 | 156 | 3 | 'TP9' | 113 |
| 28 | 156 | 3 | 'T7', 'TP9', 'TP7' | 118 |
| 29 | 166 | 3 | None | 137 |
| 30 | 164 | 3 | 'T8', 'TP9' | 114 |
| 31 | 155 | 5 | *None* | 99 |
| 32 | 163 | 3 | *None* | 121 |
| 33 | 164 | 4 | *None* | 125 |
| 34 | 149 | 2 | 'TP9' | 106 |
| 35 | 160 | 3 | 'T7' | 124 |
| 36 | 160 | 3 | 'TP8' | 134 |
| 37 | 163 | 3 | 'FP1', 'TP9' | 140 |

***Supplementary Table 1*.** Summary of preprocessing steps.

|  | **Temporal** | | | **Spectro-temporal** | | **Rating** |
| --- | --- | --- | --- | --- | --- | --- |
|  | ***C*** | ***CP*** | ***P*** | ***α*** | ***θ*** | ***Pain-NoPain*** |
| **COG** | .11  [-.23, .42] | -.01  [-.33, .32] | -.07  [-.39, .26] | .10  [-.25, .41] | .06  [-.27, .38] | .13  [-.21, .44] |
| **RES** | .20  [-.13, .50] | .23  [-.11, .52] | .19  [-.15, .49] | -.28  [-.57, .04] | .21  [-.13, .50] | .17  [-.17, .47] |
| **DIS** | .00  [-.33, .33] | -.11  [-.43, .23] | -.07  [-.39, .26] | -.03  [-.36, .30] | .10  [-.23, .42] | .07  [-.27, .39] |
| **CAL** | -.30  [-.57, .03] | -.35  [-.61, -.02] | -.22  [-.51, .12] | .21  [-.13, .50] | -.03  [-.35, .31] | -.18  [-.48, .16] |
| **UC** | -.34  [-.60, -.02] | -.36  [-.62, -.03] | -.33  [-.59, .00] | .24  [-.09, .53] | -.17  [-.48, .16] | -.12  [-.43, .22] |
| **UN** | .03  [-.30, .36] | -.18  [-.48, .16] | -.24  [-.53, .10] | .22  [-.12, .51] | .39  [.07, .64] | .03  [-.30, .36] |
| ***Supplementary Table 2.*** Correlations between pain perception and socio-affective traits*.* Temporal features refer to event-related potentials extracted from central (C), centroparietal (CP), and parietal (P) electrodes. Spectro-temporal features represent event-related spectral power changes in the alpha (α) and theta (θ) bands, only observed over CP electrodes. All correlation coefficients are presented with their 95% confidence intervals in brackets.  The behavioural variables are defined as follows: COG = Cognitive empathy, RES = Affective resonance, DIS = Affective dissonance, CAL = Callousness, UC = Uncaring behaviour, UN = Unemotional traits. “Pain-NoPain” indicates the difference in subjective pain ratings between painful and non-painful conditions. | | | | | | |


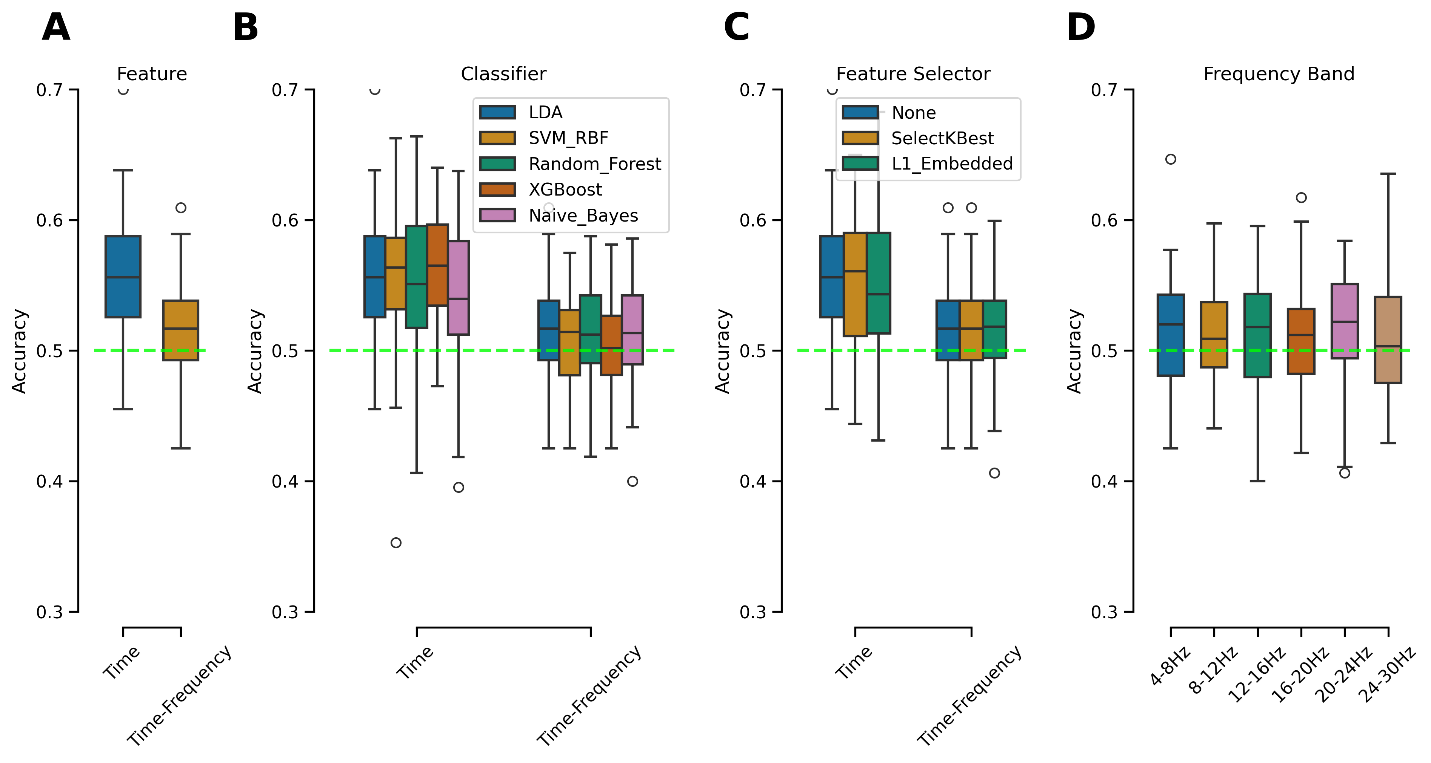


***Supplementary Figure 2*.** Assessment of classification performance across modelling parameters. Box plots illustrate classifier performance under different modelling conditions. (A) Comparison of time versus time–frequency features using a linear discriminant analysis (LDA) classifier without feature selection. (B) Effect of classifier choice (LDA, SVM-RBF, Random Forest, XGBoost, and Naïve Bayes) on accuracy across both feature types. (C) Effect of feature selection methods (None, SelectKBest, and L1-embedded) on classification performance using LDA. (D) Classification accuracy across individual frequency bands (4-30 Hz) with LDA and no feature selection. The green dashed line represents chance-level performance (50%). Box plots display the median, interquartile range, and outliers (values exceeding 1.5 times the interquartile range).


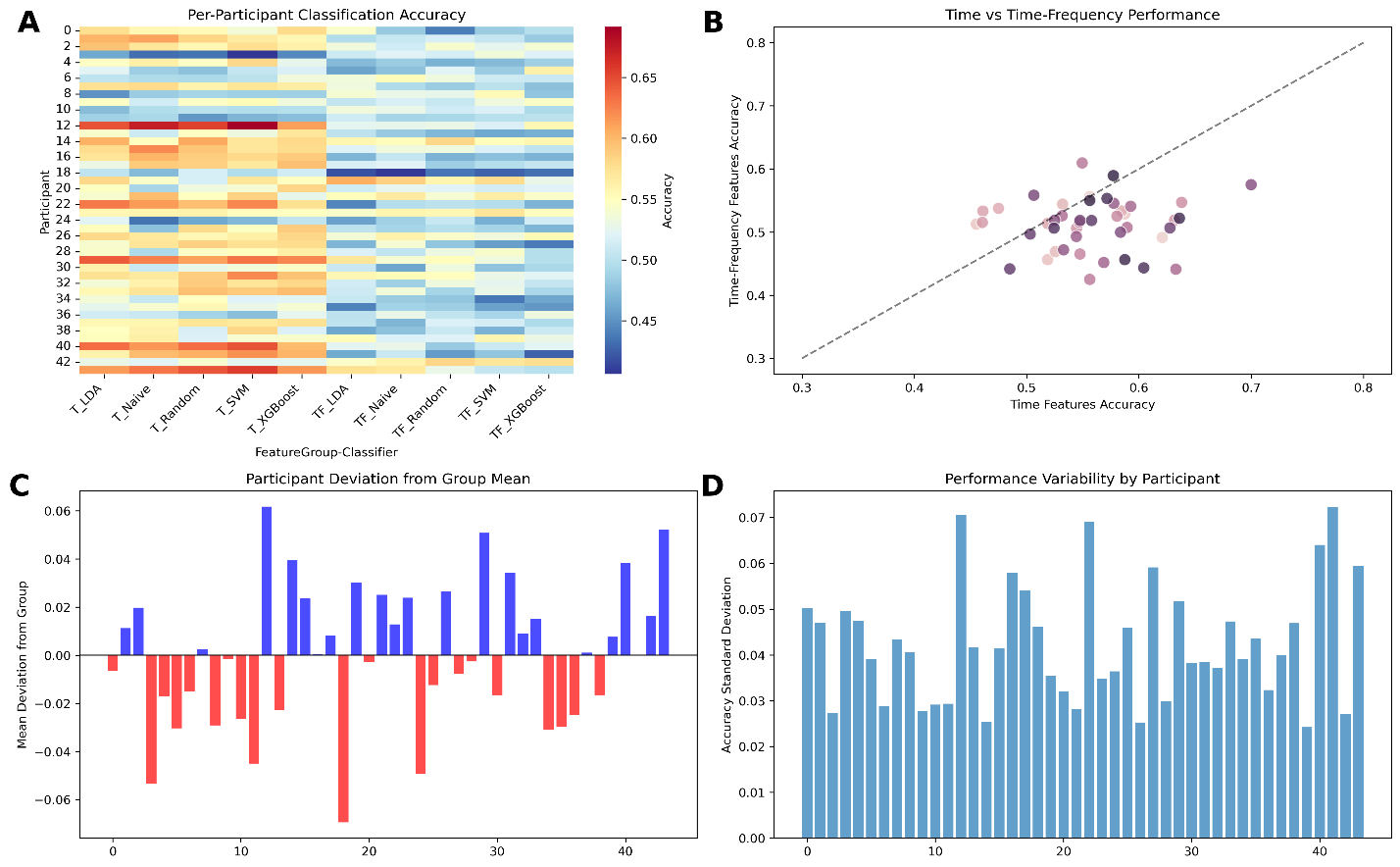


***Supplementary Figure 3.*** Individual-level classification performance. Visualisation of individual differences in classification accuracy. (A) Heatmap showing per-participant classification accuracy across feature sets (time and time–frequency) and classifiers. (B) Scatter plot comparing individual accuracies between time and time–frequency features; points above or below the diagonal line indicate relative feature-type preferences. (C) Participant deviations from the group mean accuracy, with blue bars indicating above-average and red bars indicating below-average performance. (D) Performance variability across participants, represented as the standard deviation of accuracy across classification conditions.
